# Pooled testing for SARS-CoV-2, options for efficiency at scale

**DOI:** 10.2471/BLT.20.283093

**Published:** 2021-08-13

**Authors:** Marie Reilly, Bhavna Chohan

**Affiliations:** a Department of Medical Epidemiology and Biostatistics, Nobels väg 12A, Karolinska Institutet, 171 77 Stockholm, Sweden.; b Department of Global Health, University of Washington, Seattle, United States of America.

## Abstract

Widescale testing for severe acute respiratory syndrome coronavirus 2 (SARS-CoV-2) infection is recognized as a key element of surveillance and outbreak control in the coronavirus disease 2019 (COVID-19) pandemic. The practical challenges, however, have often led to testing only symptomatic individuals and their close contacts. As many countries plan for a cautious relaxation of social restrictions, more effective approaches for widescale testing are increasingly important. Early in the COVID-19 pandemic, laboratories in several countries demonstrated the feasibility of detecting SARS-CoV-2 infection by pooled testing, which combines the specimens from several individuals. Since no further testing is needed for individuals in a negative pool, there is potential for greater efficiency of testing. Despite validations of the accuracy of the results and the efficiency in testing specific groups, the benefits of pooling are less acknowledged as a population surveillance strategy that can detect new disease outbreaks without posing restrictions on entire societies. Pooling specimens from natural clusters, such as school classes, sports teams, workplace colleagues and other social networks, would enable timely and cost-effective widescale testing for SARS-CoV-2. The initial result would be readily translatable into action in terms of quarantine and isolation policies. Clusters of uninfected individuals would be quickly identified and immediate local lockdown of positive clusters would be the appropriate and sufficient action while retesting those individuals. By adapting to the social networks of a population, pooled testing offers a cost-efficient surveillance system that is synchronized with quarantine policies that are rational, risk-based and equitable.

## Résumé

Le dépistage à grande échelle de l'infection au coronavirus 2 du syndrome respiratoire aigu sévère (SARS-CoV-2) est considéré comme l'un des piliers de la surveillance et de la lutte contre la pandémie de maladie à coronavirus 2019 (COVID-19). Néanmoins, les défis pratiques inhérents à ce dépistage ont souvent poussé à ne tester que les individus symptomatiques et leurs contacts rapprochés. Alors que de nombreux pays prévoient de lever certaines restrictions sociales, il devient de plus en plus important d'adopter des approches plus efficaces pour dépister à grande échelle. Dès le début de la pandémie de COVID-19, des laboratoires répartis dans divers pays ont démontré qu'il était possible de détecter une infection au SARS-CoV-2 par le biais de tests groupés, qui rassemblent des échantillons provenant de plusieurs individus. Si le résultat de l'un des groupes est négatif, nul besoin de tester chaque individu, ce qui pourrait accroître considérablement l'efficacité du processus. Même si les résultats se sont révélés fiables et que le dépistage de groupes spécifiques a prouvé l'efficacité du système, le regroupement d'échantillons est rarement envisagé comme stratégie de surveillance de la population permettant d'identifier toute nouvelle flambée des contaminations sans imposer de restrictions à l'ensemble de la société. Pourtant, le regroupement d'échantillons provenant de foyers épidémiques naturels tels que les salles de classe, les équipes sportives, les collègues de travail et autres interactions sociales ferait gagner du temps et de l'argent lors du dépistage à grande échelle du SARS-CoV-2. Le résultat initial pourrait aussitôt se traduire par des mesures d'isolation et de quarantaine. Les groupes d'individus non infectés seraient rapidement repérés, et le confinement immédiat des groupes positifs constituerait une intervention appropriée et suffisante pendant que ces individus subiraient un nouveau test. En s'adaptant aux interactions sociales d'une population donnée, les tests groupés représentent une solution de surveillance rentable en phase avec une politique de quarantaine rationnelle, équitable et fondée sur une analyse des risques.

## Resumen

Las pruebas a gran escala para detectar la infección por el coronavirus del síndrome respiratorio agudo grave-2 (SARS-CoV-2) se reconocen como un elemento clave de la vigilancia y el control de los brotes de la pandemia por enfermedad del coronavirus (COVID-19). Sin embargo, los desafíos prácticos han llevado a menudo a realizar pruebas solo a las personas sintomáticas y a sus contactos cercanos. A medida que muchos países planifican una cautelosa relajación de las restricciones sociales, es cada vez más importante contar con enfoques más eficaces para la realización de pruebas a gran escala. Al principio de la pandemia de COVID-19, los laboratorios de varios países demostraron la viabilidad de la detección de la infección por el SARS-CoV-2 mediante pruebas conjuntas, que combinan las muestras de varias personas. Dado que no se necesitan más pruebas para las personas en un grupo negativo, existe la posibilidad de que las pruebas sean más eficaces. A pesar de las validaciones de la exactitud de los resultados y la eficiencia en las pruebas de grupos específicos, los beneficios de la agrupación son menos reconocidos como una estrategia de vigilancia de la población que puede detectar nuevos brotes de la enfermedad sin plantear restricciones en sociedades enteras. La puesta en común de muestras procedentes de grupos naturales, como clases escolares, equipos deportivos, compañeros de trabajo y otras redes sociales, permitiría realizar pruebas a gran escala, oportunas y rentables, para detectar el SARS-CoV-2. El resultado inicial permitiría ejecutar fácilmente políticas de cuarentena y aislamiento. Se identificarían rápidamente los grupos de personas no infectadas y bastaría con una cuarentena inmediata de los grupos positivos mientras se vuelven a realizar las pruebas a dichas personas. Al adaptarse a las redes sociales de una población, las pruebas conjuntas ofrecen un sistema de vigilancia rentable que se sincroniza con políticas de cuarentena que son racionales, basadas en el riesgo y equitativas.

## ملخص

إن الفحص واسع النطاق للكشف عن العدوى بفيروس كورونا 2 (SARS-CoV-2) لمتلازمة الجهاز التنفسي الحادة، يُعد العنصر الأساسي لمراقبة والسيطرة على تفشي وباء مرض فيروس كورونا 2019 (كوفيد 19). إلا أن التحديات العملية في كثير من الأحيان قد أدت إلى فحص الأفراد الذين تظهر عليهم الأعراض فقط، والمخالطين لهم عن قرب. نظرًا لأن العديد من البلدان تخطط للتخفيف الحذر من القيود الاجتماعية، تزداد أهمية الأساليب الأكثر فعالية للفحص على نطاق واسع. في وقت مبكر من وباء كوفيد 19، أظهرت المختبرات في العديد من البلدان جدوى اكتشاف عدوى SARS-CoV-2 عن طريق الاختبار المجمع، والذي يجمع عينات من عدة أفراد. نظرًا لعدم الحاجة إلى مزيد من الفحص للأفراد الموجودين في مجموعة سلبية، فإن هناك إمكانية لزيادة فعالية الفحص. على الرغم من عمليات التحقق من دقة النتائج، والكفاءة في فحص مجموعات معينة، إلا أن فوائد التجميع تنال تقديرًا أقل على أنها استراتيجية للمراقبة السكانية، يمكنها اكتشاف حالات تفشي للأمراض الجديدة دون فرض قيود على مجتمعات بأكملها. إن تجميع العينات من التجمعات الطبيعية، مثل الفصول المدرسية، والفرق الرياضية، والزملاء في مكان العمل، والشبكات الاجتماعية الأخرى، من شأنه أن يتيح إجراء فحص واسع النطاق في الوقت المناسب وبتكلفة معقولة لـ SARS-CoV-2. ستكون النتيجة المبدئية قابلة للتأويل بسهولة إلى إجراءات من حيث سياسات الحجر الصحي والعزل. سيتم تحديد مجموعات الأفراد غير المصابين بالعدوى بسرعة، وسيكون الإغلاق المحلي الفوري للمجموعات الإيجابية الإجراء المناسب والكافي أثناء إعادة فحص هؤلاء الأفراد. يوفر الفحص المجمع، من خلال التكيف مع الشبكات الاجتماعية للسكان، نظامًا للمراقبة فعال التكلفة، يتزامن مع سياسات الحجر الصحي المنطقية والقائمة على المخاطر والمساواة.

## 摘要

广泛地开展严重急性呼吸综合征冠状病毒-2 (SARS-CoV-2) 感染检测是的新型冠状病毒肺炎 (COVID-19) 大流行监测和爆发控制的关键要素。但是，现实的困难往往导致只能对表现出症状的个人及其密切接触者进行检测。由于许多国家计划谨慎地放宽社交限制，因此更加高效的大规模检测方法变得越来越重要。新型冠状病毒肺炎疫情早期，几个国家的实验室证明了通过合并检测法来判断是否感染 SARS-CoV-2 的可行性，该方法将几个人的试样合并起来进行检测。由于无需对阴性试样组中的个体开展进一步的检测，因此该方法有可能提高检测效率。尽管结果的准确性和检测特定群体的效率已经得到了验证，但合并检测法作为一种可以在不对整个社会造成限制的前提下发现新的疾病爆发的人口监测策略，其好处却不太被承认。合并来自自然群集（如学校班级、运动队、办公场地的员工以及其他社交网络群体）的样本，将实现及时且具有成本效益的大规模 SARS-CoV-2 检测。初步检测结果将轻松转化为检疫和隔离政策方面的措施。该方案可以迅速发现未感染群集，在对这些人开展再次检测的同时，立即对阳性群集实施局部封锁，是一种恰当且充分的处理方式。通过适应人群所形成的社交网络，合并检测法提供了一种具有成本效益的监测系统，可以与基于风险制定的合理公平的隔离政策同步实施。

## Резюме

Широкомасштабное тестирование на наличие коронавирусной инфекции, приводящей к тяжелому острому респираторному синдрому (SARS-CoV-2), признано ключевым элементом надзора и контроля за вспышками заболевания в ходе эпидемии коронавирусной болезни 2019 года (COVID-19). Практические сложности, однако, часто приводили к тестированию только лиц с симптомами и тех, кто находится с ними в тесном контакте. По мере того как многие страны планируют осторожно смягчать социальные ограничения, все большую важность приобретают более эффективные подходы к широкомасштабному тестированию. В начале пандемии COVID-19 лаборатории ряда стран продемонстрировали практическую приемлемость выявления инфекции SARS-CoV-2 методом тестирования объединенных образцов, в ходе которого объединялись образцы, взятые у нескольких лиц. Так как в случае отрицательного результата для объединенного образца дальнейшее тестирование не является необходимым, этот подход потенциально может сделать тестирование более эффективным. Несмотря на валидацию точности результатов и эффективность метода в тестировании конкретных групп населения, преимущества объединения образцов пока плохо осознаются в качестве стратегии популяционного наблюдения, которая может выявлять новые случаи вспышек заболевания, не налагая в то же время ограничений на все общество. Объединение образцов, собранных в естественных кластерах, таких как классы учебных заведений, спортивные команды, коллеги по работе и другие социальные объединения, позволило бы своевременно и экономично тестировать население на SARS-CoV-2 в широких масштабах. Первоначальные результаты могли бы легко претворяться в действия, такие как карантин и политика изоляции. Кластеры незараженных лиц быстро выявлялись бы, и немедленный локдаун положительных кластеров был бы соответствующей и надежной мерой при одновременном повторном тестировании участников кластера. За счет адаптации к социальным связям в популяции тестирование объединенных образцов предлагает экономически эффективную систему надзора, которая синхронизируется с правилами карантина, построенными на принципах рационального подхода, анализа рисков и сбалансированности.

## Introduction

In the early days of the pandemic of coronavirus disease 2019 (COVID-19), testing capacity was a weakness in many countries. Severe restrictions on people’s movements seemed the only response that could match the speed of transmission of the newly identified severe acute respiratory syndrome coronavirus 2 (SARS-CoV-2). The practical challenges of conducting widescale testing and identification of the virus, and the inability of global manufacturing capacity to meet these demands, led to many countries using their available resources to test only symptomatic individuals and their close contacts. However, a large proportion of infected people have no symptoms of COVID-19,^1^^,^^2^ and the virus is most transmissible 1–2 days before symptom onset.^2^ Statistical modelling has estimated that more than half of all transmission is from asymptomatic carriers.^3^ The importance of control efforts to identify these individuals has been amply demonstrated, from contact tracing in the Republic of Korea and stringent lockdown and border control in Australia and New Zealand, to mass testing in China.^4^^–^^7^ In many countries, recognition of the importance of asymptomatic carriers in perpetuating the pandemic has led to mandatory testing (and quarantine) for international travellers, serial testing for essential workers, schoolchildren and other groups, and walk-in test centres in areas with high COVID-19 incidence. These initiatives fall short in suppressing the pandemic, as cases of community transmission continue and new outbreaks occur. 

As many countries try to implement a cautious relaxation of social restrictions, widescale testing has again become important, not just to stem the resurgence when new outbreaks of infection occur, but also to put in place more effective strategies for ongoing surveillance and control. The other issue under debate is the design and imposition of restrictions that are acceptable to society and how and when these can be relaxed without compromising what they achieve. Social behaviour needs to be tailored to the level of SARS-CoV-2 infection in the local population. Yet, although testing and social restrictions are inextricably linked, these are often discussed as separate issues by scientists, health authorities and governments. We outline in this article how current scientific knowledge and lessons learnt from the COVID-19 pandemic can inform more efficient and cost-effective surveillance strategies that synergize with targeted, evidence-based social action.

## Pooled testing 

Most countries do not try to identify all asymptomatic carriers of SARS-CoV-2 in the population, reflecting the assumption that it is simply not worthwhile to conduct polymerase chain reaction (PCR) testing of large numbers of individuals to identify a few positives. However, there are alternatives to testing millions of (likely uninfected) individuals or conducting targeted testing of close contacts, health-care workers and other high-risk groups. Pooled or group testing, also known as batch testing, combines specimens from several individuals in a single test. Pooling can enable timely and cost-effective widescale testing and can directly inform actions such as the quarantine and isolation policies that have been highlighted for their important contribution to outbreak control. The use of pooled testing for SARS-CoV-2 infection has been discussed in the context of serial testing of high-risk groups, such as those in hospitals and clinics, care homes, the army and other services^8^ and for testing in the workplace.^9^ However, the benefits of pooling are less acknowledged as a potential solution for a surveillance system that can detect and suppress new outbreaks without the need for restrictions on entire societies.

## Feasibility

The pooled testing principle has been presented in detail by many authors.^8^ Briefly, in a pool that tests negative, no further testing of individuals is required; only members of positive pools need further testing to identify the positives. Pooling was first proposed in the 1940s for syphilis testing.^10^ Later, pooling was applied to test for other infections, such as hepatitis B and C and human immunodeficiency virus,^11^ and was used for high-throughput screening of blood donors.^12^ Early in the pandemic in 2020, laboratories in various countries demonstrated the feasibility of pooling for SARS-CoV-2 testing. Scientists at the Thai Red Cross created pools of size 10 where one or two known positive specimens were combined with known negatives.^13^ Researchers in Israel demonstrated that a single SARS-CoV-2-positive sample could be detected in pools of size 32 or even larger.^14^ A study comparing the result from individual and pooled testing for more than 130 000 specimens confirmed the feasibility and efficiency of pooling for large-scale testing.^15^ A summary of the experience, successes and limitations of pooled testing for SARS-CoV-2 up to the end of March 2021 reported results from 13 countries across the world, with nasopharyngeal and oropharyngeal specimens mostly tested in pools of 4–10 samples.^8^ The enormous effort of testing the entire population of Wuhan province, China, relied on pooling specimens to improve efficiency.^4^

Pooling was initially perceived as useful for laboratories with low capacity for testing for SARS-CoV-2, as a means of coping when resources were overwhelmed by demand.^16^ However, it quickly became clear that pooling offered large gains in efficiency and costs for screening asymptomatic individuals, since most pools will test negative and thus eliminate the need for a large number of individual tests.^16^^,^^17^ The anticipated dilution effect when specimens are pooled in the laboratory has not been a serious drawback in practice, where even individual samples with a low concentration of virus could be confidently identified in pooled assays.^16^ By pooling swabs in a single tube at the point of collection, the dilution effect can be avoided.^18^

## Benefits

The implementation of pooled testing requires little extra capability in laboratories that have the capacity for PCR testing. Thus the benefits of pooling are available to low- and middle-income countries that have the technical knowledge but lack the economic resources to purchase sufficient kits and reagents for individual testing. Although the efficiency of pooling is greatest at a low prevalence of SARS-CoV-2 infection in the tested population, even for prevalences up to 10% there is an overall increase of 69% in testing capacity using pools of 10 or fewer.^19^ Thus, even the low-resource countries currently experiencing the highest disease burden can make substantial gains by pooling. The number of tests required would be at least fivefold fewer for pools of 10 than for individual testing where the prevalence of SARS-CoV-2 viral infection in the tested population is 1.0% or lower. Pooling can double the testing capacity (that is, halve the total number of tests needed) where this prevalence is up to 5%, which is one of the primary World Health Organization (WHO) indicators for a low or moderate level of community transmission.^20^ Where the prevalence in the tested population is much higher (for example, the proportion of positive tests was more than 20% in Argentina, Columbia, Indonesia and Mexico at the beginning of July 2021)^21^ the under-testing of these populations could be overcome with available resources by conducting more comprehensive testing using pools. With the evolution of several variants of SARS-CoV-2, there is concern that a change in the viral genome might lower the sensitivity of the PCR assay, which would also affect the potential efficiency from pooling.^22^ Since a test that uses just a single genetic target is vulnerable to such loss of sensitivity, the United States Food and Drug Administration now recommends that SARS-CoV-2 tests should include multiple genetic targets, and developers and scientists should continuously monitor any new genetic changes so that tests can be modified if needed.^23^

## Strategies

To identify the infected individuals in a pool that tests positive, the simplest strategy is to proceed with individual testing of all members of the pool. There are also various other ways of proceeding with this second step,^8^ but the additional planning for specimen handling may outweigh the gain in efficiency. Positive individuals can also be identified in one step if each individual is represented in more than one pool in a special configuration,^9^ but this too has greater practical complexity. A free online tool is available^24^ to calculate the expected number of tests for specific pool designs, or the optimal design to achieve an acceptable sensitivity and specificity of the final test result for a given prevalence of infection in the tested population.^19^ Here we will focus on two aspects of the final outcomes from a laboratory conducting pooled testing: (i) whether a pool tests negative or positive, and (ii) which member(s) of a positive pool subsequently test positive. We also examine how this information could be used to bridge the gap between the two arms of pandemic control: testing and social restrictions.

### Vulnerable group pools

The COVID-19 pandemic has highlighted many vulnerable groups of individuals, such as those in care homes and in prisons.^25^^,^^26^ Pooled testing is particularly suited to these environments, and there are reports of pooled testing in such settings: pools of 20 in care homes in Spain,^27^ pools of up to 10 for army and other service personnel in the United States of America (USA),^28^ and pools of eight and 16 in hospitals in Brazil and Israel.^18^^,^^29^ In addition to these groups of individuals, serious outbreaks of COVID-19 have highlighted clusters of infection in certain workplaces such as meat-processing plants. While the work environment facilitates transmission, several other factors also contribute to the risk to these workers, including socioeconomic factors and testing and tracing capacity.^30^ The well-recognized risk from crowded accommodation and crowded transport suggests how pooled testing could be used to provide an efficient surveillance tool.

### Activity-based pools

There are also many activity-based clusters of individuals, such as sports teams, where pooled testing would be a rational approach for groups of individuals who will all be subject to social restrictions on detection of any positive cases. For example, pooled testing would enable a much more efficient use of testing resources for the ongoing serial testing of players and staff in England’s Premier League football clubs, for which weekly testing results are made public.^31^ For the week of 3–9 May 2021, just one positive case of SARS-CoV-2 infection was reported among 2712 players and club staff. Pools of size 20 would have required only 156 tests to identify the single positive, and in the 3 months before, approximately 2000 tests would have sufficed instead of the almost 33 000 reported on the website. This failure to capitalize on the potential of pooled testing is also a feature of the Hong Kong Special Administrative Region initiative to test every single worker in every single restaurant with the objective of removing the threat to customers posed by asymptomatic individuals.^32^ With 200 000 workers across 16 000 restaurants, the potential efficiency of pooling is obvious, especially if this is to be a routine procedure.

### College and school testing

College students have been recognized for some time as another point-of-activity group at risk of SARS-CoV-2 transmission. The pooling of specimens in a university population would not only provide significant savings, but also focus the surveillance efforts on student groups (classes, residences or other shared spaces) at high risk. Such adaptive pooled testing enabled Duke University campus in the USA to remain open for 10 weeks of classes without a serious outbreak of infection.^17^ The Government’s operational guidance for higher education institutions in the United Kingdom of Great Britain and Northern Ireland^33^ recommends testing students and staff on their return to campus and at regular intervals thereafter. Many British universities offer testing to their students and staff using antigen tests, which have lower sensitivity than the gold standard PCR test for SARS-CoV-2, especially in asymptomatic individuals,^34^^,^^35^ and so are unsuitable for pooling. Various strategies for testing and retesting are used and the burden of testing reported as 500–1500 tests per true positive.^36^ Pooled PCR testing offers a valid result for a fraction of this effort. If all individuals are tested using pools of size 10, the tests per true positive would be approximately 20–30 if the prevalence of SARS-CoV-2 infection were 1% or lower in the university population, and the tests per true positive would be 10 or fewer for a prevalence of at least 5%. In recent months, schoolchildren are also being recognized as carriers of infection^37^ and, as with college students, children in specific classes or activity groups would be natural clusters for pooling. 

### Household pools

Households or groups of several households have been encouraged to limit their contact with others during the pandemic to reduce the number of close contacts of any individual and hence the risk of transmission. A study that simulated pooling of households and the social interactions between them concluded that the whole Belgian population of 11 million individuals could be tested within 4 weeks,^38^ similar to the achievement of Chinese health authorities in screening the 11 million residents of Wuhan province in 2 weeks in 2020.^4^

### Case-based clusters

In addition to vulnerable groups, households and activity-based clusters, natural clusters for pooling may also evolve over time, such as the dynamic case-based clusters defined by the contacts of a confirmed case. Mobile phone applications for contact tracing have been designed for the automatic detection of individuals who have been in the proximity of a confirmed case of SARS-CoV-2 infection. These people constitute a natural group for testing together, perhaps invited through the same mode of contact (such as text messaging) that informs them of their potential exposure. Tests could be first conducted using large pools, and subsequently in smaller pools based on perceived risk level, such as closeness or frequency of the contact to the known case. Networks may be updated or new networks revealed by the data that accrue from automated tracing and testing. These measures will also increase our understanding of how the behaviour in different societies changes in response to the dynamic of the COVID-19 pandemic.

### Limitations to pooling 

While there are numerous social networks for which pooled testing for SARS-CoV-2 is a rational approach, pooling would not be indicated for vulnerable or hard-to-reach populations such as homeless people or persons who inject drugs. For test centres offering a service to airline passengers, departing passengers (on different flights) do not constitute a logical cluster. In contrast, testing on arrival, pooled by flight, could reduce the imposition of mandatory hotel quarantine to just those who constitute a risk to society. Pooling would also be of questionable value for subpopulations with high mobility, who would thus belong to many networks not necessarily served by the same screening team or laboratory.

## Practical considerations

There are many practical considerations that affect the feasibility and performance of pooled testing for SARS-CoV-2, including the ease of specimen collection, obtaining a sample of sufficient volume and good quality, the extraction method of the viral nucleic acids in the laboratory, the method used for detection, and the sensitivity of the assay. The nasopharyngeal or oropharyngeal swabs that are widely used for PCR assays can be difficult and uncomfortable to collect. Other more convenient specimen types, such as saliva^39^^–^^41^ and nasal swabs,^42^ are being investigated, with encouraging results. A systematic review of the diagnostic performance of different swabs found that saliva and nasal swabs had good performance and were acceptable alternatives to nasopharyngeal swabs. Importantly, the diagnostic accuracy of testing was not reduced for self-collected specimens.^43^ The ease of self-collection and reduced need for staff and personal protective equipment make saliva specimens useful, but pooled saliva specimens are not suitable for mass screening as the dilution effect lowers the sensitivity of the assay.^39^ However, pooled results from saliva samples might still be useful for quickly identifying and isolating individuals with a high concentration of virus particles.^41^

The wastewater sample from a cluster of houses, an apartment block or an individual building is essentially a large pool representing the specimens of individuals who reside there. Many countries have investigated the potential of testing for SARS-CoV-2 in wastewater as an early outbreak warning system, with experiences reported in a scientific brief from WHO.^44^ Testing of wastewater was approved in New York State, USA, on 14 August 2020^45^ and enabled Syracuse University to implement localized quarantine of students residing in a residence hall where SARS-CoV-2 was detected in the wastewater. This immediate local action enabled containment of further spread until all individuals were tested and appropriate public health interventions were put in place. A recent publication presents the experiences of 25 college campuses in the USA with using this surveillance strategy.^46^

## Social policies

It is recognized that a high level of coordination between different decision-makers is needed to manage the containment of the COVID-19 pandemic and its consequences for society.^47^ However, the potential of synchronizing testing strategies with more effective social policies has received less attention. The debate about the effective use of testing in the early months of the pandemic^48^ has given way to political leaders being called upon to devise more equitable and fact-based policies for quarantine.^49^ The imposition of national or regional restrictions on movement and quarantine rules places an undue burden on some groups, a burden which could be mitigated by pooled testing and other surveillance strategies such as wastewater testing. Many countries have faced challenges with enforcement of restrictions but, without proof of infection status, it is difficult to modify the behaviour of those who perceive the lockdown as an infringement of their liberty. With the high transmissibility of SARS-CoV-2, even a small subpopulation can contribute to maintaining an endemic infection and endless social restrictions for the wider society. Using natural social networks to guide widescale pooled testing could directly inform flexible, risk-based social policies concerning quarantine and isolation that are seen as rational and fair. The demand for over-the-counter antigen testing kits suggests that the public would be amenable to a testing strategy that focused on their household, social group, workplace or other network as a logical cluster. Since the individuals in a pool will also understand that they will be subject to the same relaxation or reintroduction of restrictions, this strategy can result in more cautious behaviour. Individuals will have a clear social responsibility in keeping their own pool of contacts free from infection, which in turn can contribute to enhanced control of the pandemic in their community.

## Conclusion

With the proven role of asymptomatic carriers in new outbreaks, it is time for health authorities, actively supported by government, to progress from targeted to mass testing. Now that pooled testing is being authorized, developers of diagnostic tests for SARS-CoV-2 are being encouraged to validate performance in pooled samples.^50^ We believe a pooling strategy adapted to social networks offers a cost-effective strategy to enable the many uninfected individuals to continue their functions in society, while lockdown restrictions are imposed only on the few who are potentially infected. This integration of testing and quarantine strategies is needed to win the public’s trust and cooperation in these challenging times.
